# The molecular genetic mechanism of the inverted allele of the human‐specific *CHRFAM7A* gene

**DOI:** 10.1002/alz70861_108411

**Published:** 2025-12-23

**Authors:** Anna Marie Szombathy, Ivanna Ihnatovych, Ryu Platinum Dorn, Eduardo Cortes Gomez, Wang Jianmin, Kinga Szigeti

**Affiliations:** ^1^ University at Buffalo, Buffalo, NY USA; ^2^ Roswell Park Cancer Institute, Buffalo, NY USA

## Abstract

**Background:**

Human genetic background defines the cellular milieu for health and disease. *CHRFAM7A* is a human‐specific fusion gene comprised of a fusion between *CHRNA7* (alpha 7 nicotinic acetylcholine receptor) and *ULK4* (an ULK kinase). This gene is found in 99% of the human population and has robust associations with many neuropsychiatric disorders, including Alzheimer’s disease and schizophrenia. The gene can be found in either direct or inverted orientations (both of which are equally prevalent in the population), but only the direct orientation is translated and incorporated into the α7 nicotinic acetylcholine receptor (α7NAChR). The mechanism of the inverted allele, meanwhile, remains uncertain.

**Method:**

We used postmortem human brain RNAseq and human isogenic iPSC derived neuronal progenitors to compare *CHRNA7* expression in inverted‐null versus inverted *CHRFAM7A* tissue and cell lines. Human samples were genotyped by locus specific TaqMan and capillary sequencing. We conducted immunoprecipitation studies with anti‐CHRFAM7A and anti‐α7NAChR antibodies to detect translation of *CHRFAM7A* and *CHRNA7* in null versus inverted iPSC lines. qPCR and western blots were used to characterize ULK4 isoforms and splicing in the inverted genetic background.

**Result:**

We found that *CHRFAM7A* expression did not affect *CHRNA7* gene expression. *CHRFAM7A* was not translated in the inverted line, and translation of *CHRNA7* did not differ between the null and inverted genotypes, suggesting that the inverted allele does not regulate *CHRNA7*. Genome browser bioinformatic analysis of *CHRFAM7A* revealed a 19bp sequence of the inverted allele which aligns with exon 13 of the *ULK4* gene on chromosome 3, suggesting targeting of *ULK4*. We found that the inverted allele displays skewing of long to short isoform ratio, with the inverted cell line demonstrating higher expression of the long isoform of *ULK4*.

**Conclusion:**

These results suggest genetic epistasis between inverted *CHRFAM7A* and *ULK4* leading to skewing of short and long isoform ratio of ULK4. The long isoform harbors heat repeats that facilitate membrane localization. The regulatory mechanism might be through RNA interference via a hairpin structure involving the 19bp targeting sequence. Improved understanding of this inverted RNAi mechanism will provide context for why the inverted allele is associated with neuropsychiatric disease.

